# The complete plastid genome sequence of the parasitic green alga *Helicosporidium *sp. is highly reduced and structured

**DOI:** 10.1186/1741-7007-4-12

**Published:** 2006-04-21

**Authors:** Audrey P de Koning, Patrick J Keeling

**Affiliations:** 1 Department of Botany, University of British Columbia. 3529-6270 University Blvd. Vancouver, BC, V6T 1Z4, Canada

## Abstract

**Background:**

Loss of photosynthesis has occurred independently in several plant and algal lineages, and represents a major metabolic shift with potential consequences for the content and structure of plastid genomes. To investigate such changes, we sequenced the complete plastid genome of the parasitic, non-photosynthetic green alga, *Helicosporidium*.

**Results:**

The *Helicosporidium *plastid genome is among the smallest known (37.5 kb), and like other plastids from non-photosynthetic organisms it lacks all genes for proteins that function in photosynthesis. Its reduced size results from more than just loss of genes, however; it has little non-coding DNA, with only one intron and tiny intergenic spaces, and no inverted repeat (no duplicated genes at all). It encodes precisely the minimal complement of tRNAs needed to translate the universal genetic code, and has eliminated all redundant isoacceptors. The *Helicosporidium *plastid genome is also highly structured, with each half of the circular genome containing nearly all genes on one strand. *Helicosporidium *is known to be related to trebouxiophyte green algae, but the genome is structured and compacted in a manner more reminiscent of the non-photosynthetic plastids of apicomplexan parasites.

**Conclusion:**

*Helicosporidium *contributes significantly to our understanding of the evolution of plastid DNA because it illustrates the highly ordered reduction that occurred following the loss of a major metabolic function. The convergence of plastid genome structure in *Helicosporidium *and the Apicomplexa raises the interesting possibility that there are common forces that shape plastid genomes, subsequent to the loss of photosynthesis in an organism.

## Background

Plastids are organelles found in plants and algae. Plastids originated in the endosymbiotic uptake of a cyanobacterium, which was subsequently transformed from a complex free-living bacterium to the highly specialized organelle now integrated with its host. At the genomic level, this integration involved the loss of many genes and the transfer of many more to the host nuclear genome, the protein products of which are targeted back to the organelle [1]. This process is not complete, however, as all known plastids have retained a residual genome that encodes a handful of RNA and protein-coding genes, which typically include many of the key components of photosystems I and II [2]. Our concept of plastids is inexorably tied to photosynthesis, since this is the dominant metabolic process of most plastids. They are, however, metabolically diverse organelles that play a role in the biosynthesis of amino acids, fatty acids, isoprenoids and heme, as well as in other processes related to photosynthesis such as pigment biosynthesis, and radical detoxification. Indeed, in several lineages of plants and algae photosynthesis has been lost altogether, but the plastid has been retained for these and other purposes [3]. Well-known examples of this include holoparasitic plants, many lineages of heterotrophic algae and parasitic apicomplexans (such as the malaria parasite). In most plastid genomes, the vast majority of genes encode products involved in either gene expression or photosynthesis. When photosynthesis is lost, so are most or all of the related genes, leading to dramatic changes in the plastid genome in size, coding capacity, and often also structure.

These genomes offer an opportunity to study the effects of massive genomic changes following a functional shift. Unfortunately, the number of fully-sequenced non-photosynthetic plastid genomes is small, limited to *Epifagus virginiana *(a holoparasitic angiosperm), *Euglena longa *(a heterotrophic euglenid), and several apicomplexan parasites bearing secondary plastids of red algal origin called apicoplasts (*Plasmodium falciparum*, *Theileria parva*, *Eimeria tenella *and *Toxoplasma gondii*). The *E. virginiana *plastid is about half the size of typical angiosperm plastids, having lost all its photosynthetic genes, but is otherwise similar to its relatives in many ways including non-coding DNA content, synteny of remaining genes and overall structure [4]. Likewise, *E. longa *has lost most of the photosynthetic genes found in the plastid of its close relative *Euglena gracilis*, but they share many features that are unique to euglenids, such as three tandem repeats of the RNA operon and a multitude of distinctive introns [5]. Apicomplexan plastid genomes, however, are quite different from those of other secondary red algal plastids found in photosynthetic lineages. They have undergone extensive rearrangments, are exceedingly small (~35 kb) and compact, and contain very little non-coding DNA [6-9]. The uniqueness of apicomplexan plastid genomes may simply be due to time: apicomplexan plastids probably lost photosynthesis long ago in the ancestor of this diverse group, whereas other sequenced non-photosynthetic plastid genomes come from organisms with close relatives that are photosynthetic.

To examine the process of genome reduction after the loss of a major metabolic function, we have completely sequenced the genome of the non-photosynthetic plastid of *Helicosporidium *sp., a parasitic green alga. Helicosporidia are obligate parasites of invertebrates with a unique morphology and infection strategy [10]. Their evolutionary origin was disputed until recently, when molecular evidence surprisingly showed that they are highly adapted trebouxiophyte green algae, specifically related to the opportunistic vertebrate parasites, *Prototheca *[11,12]. This led to the prediction that Helicosporidia contain plastids, and although they have not yet been visually identified, molecular evidence has confirmed their existence [13-15]. The function of this organelle has been investigated by examining nucleus-encoded plastid-targeted proteins, which cumulatively suggest the *Helicosporidium *plastid is functionally similar in many ways to that of apicomplexan parasites [15]. Here, we show that the *Helicosporidium *plastid genome, while retaining many features confirming its phylogenetic affiliation, has been radically reduced in a non-random, structured fashion. The result is a genome that is highly ordered with regard to several characteristics such as coding strand and selective loss of tRNAs. Comparing the *Helicosporidium *plastid genome to that of other green algae and more distantly related non-photosynthetic plastids raises the interesting possibility that the 'structured-reduction' of *Helicosporidium *represents a common fate of such a genome.

## Results and discussion

### Genome size and density of coding regions

**Figure 1:**
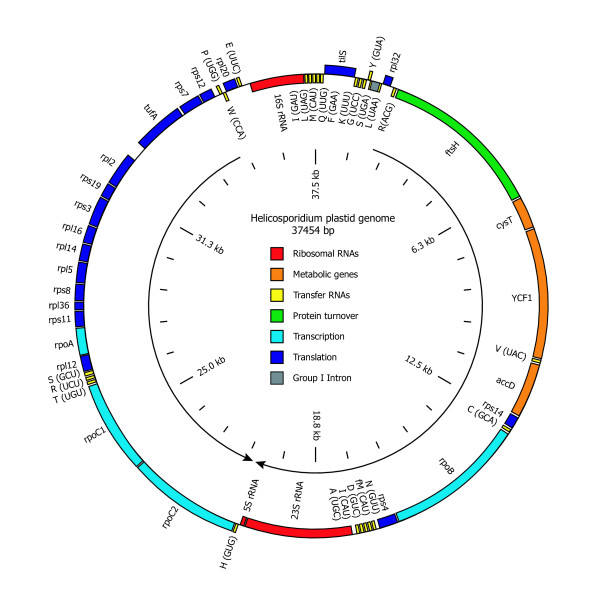
**Gene map of *Helicosporidium *plastid DNA**. Genes on the inside of the map are transcribed clockwise, while those on the outside are transcribed counter-clockwise. Transfer RNAs are indicated by the one-letter amino acid code followed by the anticodon in parentheses. rRNA: ribosomal RNA subunits, *rpo*: RNA polymerase subunits, *tufA*: elongation factor Tu, *tilS*: tRNA(Ile)-lysidine synthetase, *ftsH*: ftsH protease, *cysT*: sulfate transport protein, *ycf1*: conserved plastid protein of unknown function, *accD*: acetyl-CoA carboxylase beta subunit.

The *Helicosporidium *sp. plastid genome was determined to be a circle 37,454 bp in length with a gene map as shown in Figure 1. It has an overall GC content of 26.9%, which is less than most plastids, but not as extreme as the 13.1% observed in the *Plasmodium falciparum *apicoplast [9]. Non-coding regions are more AT-rich (14.7% GC) than genes, and are small. Gene-density is high, with only 5.1% non-coding DNA and an average intergenic space of 36 bp. Four gene pairs overlap by between 4 and 27 bp. *Helicosporidium *has by far the smallest plastid genome of any known Viridiplantae (plants and green algae), and is the smallest sequenced plastid genome outside those of apicomplexan parasites. One of the most compact plastid genomes reported so far is the primitive red alga *Cyanidioschyzon merolae*, which although extremely gene rich was reported by Ohta *et al*. [16] to have a median intergenic distance of just 14 bp. Using the same measure of compactness as Ohta *et al*. (the median of intergenic spaces between adjacent protein coding genes, where overlapping genes have a negative intergenic space), *Helicosporidium *is comparably compact with a median intergenic distance of 8 bp.

A comparison of the genomic features of non-photosynthetic plastids and their photosynthetic relatives is presented in Table 1. Compared with the photosynthetic trebouxiophyte *Chlorella vulgaris*, the *Helicosporidium *plastid has undergone a 4-fold reduction in genome size through large scale gene loss (4-fold), compaction of the remaining genes with smaller intergenic regions (7-fold) and an overall lower proportion of non-coding sequence (3.7-fold). The opportunistic parasite *Prototheca wickerhamii *is a close relative of *Helicosporidium *[11,12,14], and has genome characteristics intermediate to *Helicosporidium *and *C. vulgaris*. At an estimated 54 kb [17], the *P. wickerhamii *plastid is one third the size of the *C. vulgaris *plastid with less non-coding DNA and more densely packed genes, but is reduced to a much lesser extent than *Helicosporidium*. Comparing other non-photosynthetic plastid genomes with photosynthetic relatives reveals that the reduction and compaction of *Epifagus virginiana *and *Euglena longa *are not as substantial (about a 2-fold reduction in size). Plastids of red algae and their derivatives tend to have more genes than those of green plastid lineages [1,2], so it is interesting that the smallest and most compact genomes are found among the red plastids of apicomplexa. A sister group comparison is difficult for this group, since the closest relatives of apicomplexa are dinoflagellates, the plastid genomes of which are difficult to compare with other plastids because they have been transformed into single gene mini-circles [18]. However, the photosynthetic ancestors of apicomplexa were probably similar to other secondary red plastid-containing organisms, such as *Odontella sinensis *(Table 1), which would indicate a 4-fold reduction in plastid genome and an even more extreme level of compaction.

### Genome structure and organization

Unlike most plastid genomes, the *Helicosporidium *genome does not contain an inverted repeat (Figure 1). Although inverted repeats are probably an ancestral character state for all plastids, they have been independently lost in several lineages. Among the green algal plastids investigated so far, the inverted repeat is absent in charophytes (*Staurastrum puctulatum *and *Zygnema circumcarinatum *[19]), ulvophytes (*Caulerpa sertularoides *[20] and *Codium fragile *[21]) and the trebouxiophyte *Chlorella vulgaris *[22], but is present in *Chlorella ellipsoidea *[23]. More interestingly, *Helicosporidium *has also lost the ribosomal RNA (rRNA) operon, which is a nearly universal feature of all genomes, including prokaryotes, eukaryotes and organelles. The plastid rRNA operon is normally part of the inverted repeat when it is present, and consists of the small and large subunit (SSU and LSU) rRNA genes separated by a spacer region containing the tRNA-Ile and tRNA-Ala genes. In *Helicosporidium*, the rRNA genes are separated by 22.6 kb of sequence, but tRNA-Ile and tRNA-Ala genes remain associated with the SSU and LSU genes, respectively, such that a typical rRNA operon has been broken in half and distributed at opposing ends of the circle (Figure 1). While the vast majority of plastids have the rRNA operon, it has been lost in *C. ellipsoidea, S. punctulatum *and the *P. falciparum *and coccidian apicoplasts, where the SSU and LSU rRNA genes are adjacent to each other but encoded on opposite strands [6,7,9,19,23]. It has also been disrupted in the charophytes, *Z. circumcarinatum *[19] and *Spirogyra maxima *[24], and the ulvophytes *C. sertularoides *[20] and *C. fragile *[21], where the two rRNA genes are located on the same strand but far apart, as in *Helicosporidium*. This genome rearrangement has therefore occurred in at least three independent lineages, and may be an outcome of loss of the inverted repeat.

The most striking feature of the *Helicosporidium *genome is the symmetry shown in strand bias of coding regions (Figure 1). The rRNA genes are nearly diametrically opposed, and all but two proteins and one tRNA on one side of them are on the same strand, while all but one tRNA on the other side are on the opposite strand. Similarly-organized coding strand biases are also found in some apicomplexan plastids and in the euglenid plastids, but the bias is not as strong. In *P. falciparum*, the coding strand switch occurs between the adjacent inverted repeats, each of which encodes LSU and SSU rRNA and nine tRNA genes [9] and contains the origin of replication [25]. In *Euglena*, the coding strand symmetry is bound on one end by the origin of replication and on the other end by the replication termination site [26]. In these organisms, the majority of genes are transcribed in each direction away from the bidirectional origin of replication, such that the leading strand of replication is largely the coding strand. Such bias is also widespread among bidirectional replicating prokaryote genomes, where it is hypothesized to be the result of selection to minimize collision between DNA and RNA polymerases moving in opposite directions [27].

**Figure 2:**
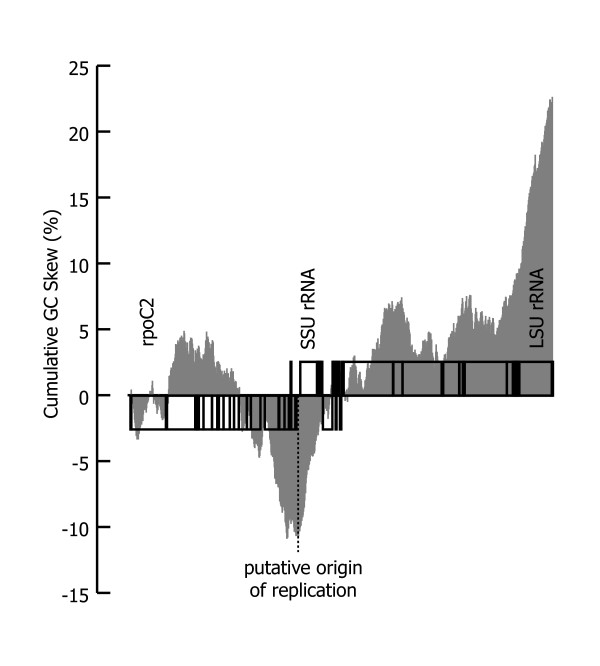
**GC skew diagram for the *Helicosporidium *plastid genome**. The sum of [G-C]/[G+C] calculated in 37 base, non-overlapping adjacent windows is shown superimposed on a linearized scale map of the genome, starting between the *LSU *and *rpoC2 *genes. The global minimum of this plot corresponds to the proposed origin of replication.

Notably, one of only two non-coding regions larger than 100 bp in the *Helicosporidium *plastid genome is situated between the SSU rRNA and tRNA-Glu genes, at one of the crossover points in strand selection. To investigate whether this could be a replication origin, we constructed a sliding window of cumulative GC skew [G-C/G+C]. These plots detect changes in compositional bias of guanine over cytosine along a sequence, which presumably occur because of strand-specific mutational biases during replication, and their global minimum and maximum points correspond to the origin and termination of replication for genomes with bi-directional replication origins [28,29]. The cumulative GC-skew of the *Helicosporidium *genome reveals a global minimum and maximum at the regions either side of the SSU and LSU genes (Figure 2B), lending support to the idea that the origin of replication is located as marked in Figure 2. If this is so, the *Helicosporidium *plastid is like those of apicomplexans and euglenids in that almost all genes are encoded on the leading strand of replication, and that the observed coding strand symmetry is an adaptation for co-directional replication and transcription. Interestingly, this bilateral symmetry is not seen in most other plastid genomes, even though the selection pressure should be universal for bi-directionally replicating circular genomes. However, other selective pressures might also increase coding strand bias. A recent examination of plastid gene order in green algae and plants showed that in the *Chlamydomonas reinhardtii *plastid genome, adjacent genes were more often functionally related and clustered on the same strand than in an inferred ancestral genome, and that this clustering is significantly higher than would be expected from random genome rearrangements [30]. At least in the highly re-arranged plastid genome of *C. reinhardtii*, and perhaps more generally, increased coding strand bias seems to be an outcome of selection for co-transcription of genes of common function.

**Figure 3:**
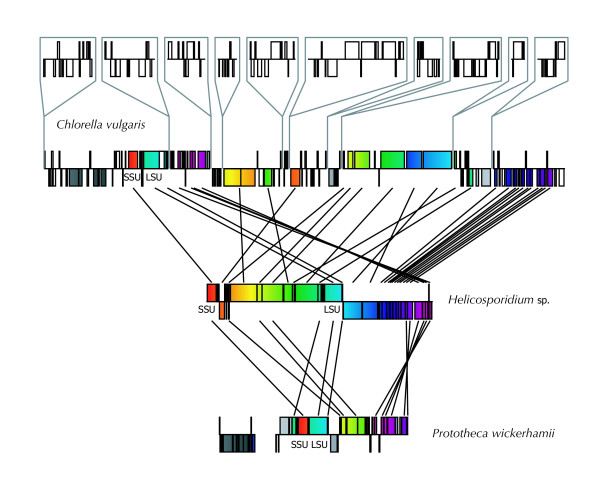
**Gene order comparisons between trebouxiophyte plastid genomes**. Genomes are drawn to scale. Coloured boxes represent identity with genes found in *Helicosporidium *sp., grey-shaded boxes represent identity between genes found in *Chlorella vulgaris and Prototheca wickerhamii*, and open boxes represent genes found only in *C. vulgaris*. Segments of the *C. vulgaris *genome that have been completely lost in *Helicosporidium *are shown in call-out boxes above the remainder of the genome. Straight lines join the genomes at the center points of protein coding and rRNA genes. For orientation, the rRNA genes are indicated.

Comparing gene order with *C. vulgaris *reveals that, in addition to the large-scale changes in genome structure, smaller rearrangements have also been common (Figure 3). The most obvious differences between the genomes are the many large deletions in *Helicosporidium*. These missing segments encode mostly photosynthetic products, but also *clpP *protease, the cell division proteins *minD *and *minE*, and several tRNAs and ribosomal proteins. In the remaining shared segments, synteny is low between *Helicosporidium *and *C. vulgaris*. Some conserved blocks do remain, such as a large string of co-directionally transcribed ribosomal proteins including L2, S19, S3, L16, L14, L5, S8, L36, S11 and RNA polymerase subunit A (*rpoA*). This particular block of genes is conserved, with some lineage-specific deletions, in all plastids and is probably co-expressed. The partial plastid genome of *P. wickerhamii *shows considerable rearrangement of genes when compared to either *C. vulgaris *or *Helicosporidium*, suggesting rapid and ongoing rearrangements in these genomes.

### Gene content

The *Helicosporidium *genome encodes 26 proteins, 3 rRNAs and 25 tRNAs. The only intron is a group I intron in the tRNA Leu (UAA) gene. This particular intron is commonly found in cyanobacteria and plastids and may be an ancestral plastid feature, although lineage-specific losses have occurred among green algae [31]. No unique ORFs of appreciable size were found and most of the protein-coding genes in *Helicosporidium *are identifiable as housekeeping proteins involved in transcription and translation (Figure 1). These include 16 ribosomal proteins, an elongation factor and components of an RNA polymerase (rpo). In plants, this polymerase is responsible mainly for transcription of genes associated with photosynthesis. In non-photosynthetic plants, algae and apicomplexans [4,5,9], some or all of the rpo subunits have been lost from the plastid genome, and it is thought that a separate nuclear-encoded polymerase is responsible for plastid transcription. In the *Helicosporidium *plastid, however, all 4 subunits of the RNA polymerase complex (*rpoA*, *rpoB*, *rpoC1*, and *rpoC2*) are present. The *Helicosporidium *plastid also encodes tRNA(Ile)-lysidine synthetase (*tilS*), which is responsible for modifying the CAU anticodon of a unique tRNA that is cognate for isoleucine. This CAU-reading tRNA is universally found among bacteria and plastids [32]. In plastids, however, *tilS *is generally encoded in the nuclear genome and targeted to the organelle. In addition to *Helicosporidium*, *tilS *is also plastid-encoded in the rhodophyta, and in the green algae *Nephroselmis olivacea*, *C. vulgaris*, *Chaetosphaeridium globosum *and *Mesostigma viride*.

Only four protein coding genes encode products not involved in transcription or translation: FtsH protease, which degrades membrane-bound proteins [33,34], *ycf1*, a poorly conserved gene of unknown function that has been shown to be essential [35,36], Acetyl-CoA carboxylase beta subunit (*accD*), which is involved in fatty acid biosynthesis [37], and a sulfate transport protein (*cysT*) [38]. These four genes have a scattered distribution among plastid genomes. FtsH protease is found in red algae, chromists (algae with plastids of secondary red algal origin) and green algae, but is nuclear-encoded in plants. *AccD *is found in plants, green algae and red algae. *Ycf1 *is only found in the plastid genomes of plants and green algae, while *cysT *is restricted to green algae, a few lower plants and one red alga. Other components of the metabolic pathways in which *accD *and *cysT *participate are known to be encoded in the nucleus of *Helicosporidium *and targeted to the plastid, confirming these as metabolic functions of the organelle [15]. As expected, no genes involved in photosynthesis or bioenergetic processes were found.

As noted earlier, *P. wickerhamii *probably represents an intermediate form between autotrophic, *Chlorella*-like trebouxiophytes and the highly-reduced *Helicosporidium*. Over half the *P. wickerhamii *plastid genome is known, and no photosystem, electron transport or chlorophyll biosynthesis proteins have been found. However, the *P. wickerhamii *plastid does encode genes for six of the subunits of ATP synthase [17], which are not present in *Helicosporidum *or apicoplast genomes.

### The *Helicosporidium *plastid encodes a minimal set of tRNAs

**Figure 4:**
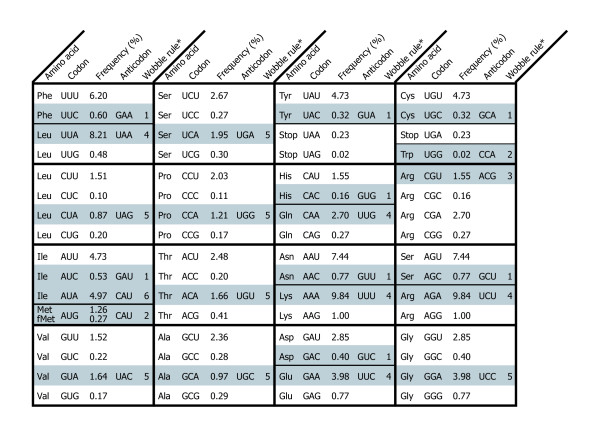
**Codon frequency and tRNAs in the *Helicosporidium *plastid**. *Wobble rules indicate the allowed mismatches between the first position of the anticodon and the third position of the codon (other positions, indicated by 'X', follow standard Watson-Crick base-pairing rules): 1) GXX anticodons read XXU and XXC codons, 2) CXX anticodons read XXG codons 3) AXX anticodons (where A is modified to I) read XXN codons, 4) UXX anticodons (where U is modified) read XXA and XXG codons, 5) UXX anticodons read XXN codons, and 6) LAU anticodons (where C is modified to L) read AUA [32, 39–41].

The *Helicosporidium *plastid genome contains just 25 tRNAs, which is among the smallest number of tRNA genes documented to date in a plastid genome (Table 1). This is in part due to a reduction in tRNA gene copy number, such that the *Helicosporidium *plastid encodes only a single copy of each tRNA with a particular anticodon. Multiple tRNA gene copies are universally found in plastids, sometimes independently (as in *C. vulgaris*) and sometimes as part of the inverted repeat (e.g *P. falciparum*). Moreover, the *Helicosporidium *plastid genome contains a minimal functional set of tRNAs for a genome using all 61 sense codons and the universal genetic code. The set of tRNAs in *Helicosporidium *is a good illustration of the degree of order in the reduction of this genome. There are twenty amino acids and each is represented by a single tRNA except leucine, serine, arginine, methionine and isoleucine (Figure 4). Leucine, serine, and arginine are distinguished by having six codons and so require two tRNAs: one for four codons and another for the other two. Methionine has a single codon, but requires an initiator tRNA and a second one for internal methionine codons. Isoleucine, with three codons, requires 2 tRNAs: one for a pair of codons ending in a purine (R) and a second that is the substrate for tRNA(Ile)-lysidine synthetase, which modifies C in the first position of the anticodon to lysidine (L), making it cognate for the codon AUA. Conceptually, this is a minimum complement of tRNAs for plastids: one each for the twelve 2-fold degenerate codon groups, one each for the eight 4-fold degenerate codon groups, one tryptophan, one initiator methionine, one elongation methionine, one for the AUR pair of isoleucine codons, and the modified tRNA-Ile.

In general, plastids use more than the minimal set: about 32 different tRNA species are usually found because more than one isoacceptor is often used to decode the 4-fold degenerate groups of serine, leucine, threonine, arginine and glycine and the 2-fold degenerate lysine (UAR) group. *Helicosporidium *minimizes the number of isoacceptors used, by complete utilization of 3^rd ^position wobble. As Figure 4 shows, the complement of tRNAs encoded in the *Helicosporidium *genome are sufficient to decode all codons found in the mRNA, assuming that some known modifications [32,39] are used. Furthermore, the GXX tRNA is present in every one of eight codon pairs ending in a pyrimidine, the UXX tRNA is present in every one of five codon pairs ending in a purine, and the UXX tRNA is present in seven out of eight 4-fold degenerate groups. The single exception to this uniformity is tRNA-Arg (ACG). The first anticodon position A is presumably modified to inosine and reads all four Arg codons, as happens in plant plastids [40,41].

Once again, the closest comparison for this type of reduction lies in the non-photosynthetic plastids. *P. falciparum *takes near-complete advantage of the wobble rules but uses two anticodons for glycine. *E. tenella, T. gondii *and *T. parva *have dispensed with the extraneous tRNA-Gly and use the same suite of tRNAs as *Helicosporidium*. Curiously, however, all the apicomplexa appear to lack the modified tRNA-Ile (CAT reading ATA). The ATA codon frequently appears in these genomes, so either a unique and unknown modification system must exist [42], or they import a tRNA. In the holoparasitic plant *E. virginiana*, a number of tRNAs have been lost or exist as pseudogenes. Seven essential anticodons are missing, so it has been suggested that *E. virginiana *must import tRNAs [43]. *Helicosporidium *is therefore unique in that it reduced its tRNA complement to a minimum without inventing new modifications, changing its genetic code or importing tRNAs from the cytoplasm; instead, it has simply done away with all redundant tRNAs to leave the perfect minimal set for the universal code.

Interestingly, there is a strong AT codon bias in *Helicosporidium *protein coding genes (the arginine codon CGG is used only once in the entire genome), and this bias is often counter to the tRNA complement (Figure 4). In some systems, there is a correlation between codon bias and what tRNA genes are present in the genome, and this is assumed to occur by selection for increased translation effieciency [32]. However, in *Helicosporidium*, codon bias is clearly a result of an overall high AT bias, while the presence or absence of tRNAs is dictated by wobble rules.

## Conclusion

When a major metabolic shift occurs, many genes may be lost. If photosynthesis disappears, this loss of genes can represent a large proportion of the plastid genome, so the effect is severe. By itself, however, this loss does not explain the nature of these reduced genomes, because there is no obvious reason that the resulting genome could not be similar in form to its photosynthetic ancestor, but reduced in content. Indeed, this is what we see in the holoparasitic plant *E. virginiana *and the heterotrophic euglenid *E. longa*. However, the genomes of the apicoplast and *Helicosporidium *are different; these genomes are highly reduced but more ordered than their ancestors. At least some aspects of this 'structured reduction' appears to be related to high coding density: the symmetrical coding strand bias probably developed to co-ordinate transcription and replication, and the elegant utilization of wobble rules is probably to reduce the complement of tRNA genes to a minimal functional set. The apicomplexa and *Helicosporidium *are not closely related; indeed, among plastid types they could hardly be more different: the apicoplast is a secondary plastid derived from a red alga whereas the *Helicosporidium *plastid is a primary green algal plastid. Their genomes have both retained characteristics that betray these origins, but they have also converged on a similar form in many ways. It is possible there are specific selective pressures operating here that are not important to other sequenced non-photosynthetic plastid genomes, or it could be that this is a predictable outcome for the evolution of these genomes, and less ordered examples are simply not there yet. Either way, the overall forms of apicomplexan and *Helicosporidium *plastid genomes have been shaped in parallel by common evolutionary forces. Comparing them raises interesting questions about whether there are selective pressures that lead genomes to compact, or if compaction is simply a by-product of reduction that occurs for neutral reasons.

## Methods

### Cell culture and genomic DNA isolation

*Helicosporidium *sp. (ATCC 50920, isolated from the blackfly *Simulium jonesii*) was cultured axenically in TNM-FH insect medium (Sigma-Aldrich) supplemented with 5% fetal bovine serum and 50 mg/ml of gentamycin at 25°C in the dark. Cells were harvested by centrifugation and ground under liquid nitrogen. Total genomic DNA was extracted from the ground cells using the Plant Dneasy Mini Kit (Qiagen).

### Genome sequencing

Genes encoding accD and cysT were amplified by PCR using the degenerate primer pairs GGCGTGATGGACTTYCANTTYATGG/GCCGTCACCCCNCCNGTNGTNG and GACTACTATGTGGAYYTNCCNTTYGC/GCCCCGAAGTARTCRTAYTGYTC, respectively. In addition, a fragment containing a portion of the rpoC1 and rpoC2 genes was sequenced as part of an ongoing genomic sequence survey, and two sequences (a partial SSU rRNA [GenBank:AF538864] and *rps12*/*rps7*/*tufA*/*rpl2 *[GenBank:AY498714]) were characterized previously. These four sequences were used as anchors for long-range PCR containing 1 U Elongase polymerase mix (Invitrogen), 1.5 mM [Mg ^2+^] buffer, 100 ng template DNA, 200 μM dNTPs and 0.2 μM each of two primers, resulting in eight overlapping fragments of the plastid genome ranging in size from 867 to 8168 bp. These fragments were TOPO TA (Invitrogen) cloned and sequenced using BigDye terminator chemistry (ABI) on both strands by primer walking. Sequences were assembled using Sequencher (Gene Codes Corporation), yielding a circular molecule, with a total of about 2.4 kb of overlap between clones. The plastid genome sequence has been deposited in GenBank [DQ398104].

### Annotation and analyses

Protein coding genes were initially identified by BLASTX [44] searches of NCBI protein databases. In cases of divergent sequence and/or length heterogeneity such that the *Helicosporidium *ORF could be defined by more than one initiation codon, the longest non-overlapping ORF was selected. Ultimately, 94.9% of the genome contained ORFs with detectable homologues in other plastid genomes, giving high confidence that all genes were identified and annotated. Ribosomal RNA (rRNA) genes were identified by BLASTN searches against the plastid genome database at NCBI. Endpoints of rRNA genes were determined by alignment with trebouxiophyte plastid rRNA genes [GenBank:AJ222802, GenBank:NC001865]. Transfer RNA (tRNA) genes were identified using tRNAscan-SE [45]. All non-coding regions were re-analyzed with BLASTX and BLASTN searches, revealing no detectable matches. Mean intergenic distances were calculated from intergenic spaces between all genes, with overlapping genes given a value of 0.

## Authors' contributions

APD designed and performed the study, interpreted the data and drafted the manuscript. PJK conceived of the study, contributed insight into data interpretation and helped to draft the manuscript. All authors read and approved the final manuscript.

## Figures and Tables

**Table 1:** Plastid genome features compared between non-photosynthetic plastids and photosynthetic relatives

	Size (kb)	% non-coding DNA (including introns)	Mean intergenic distance (bp)	No. of genes (proteins & rRNAs)	No. of tRNAs	Large repeat	Accession No.
**Trebouxiophytes**							
*Helicosporidium *sp.	37.5	5.1	36	29	25	0	[DQ398104]
*Prototheca wickerhammii*	*54.1	21.4	121	-	-	-	[AJ245645, AJ236874]
*Chlorella vulgaris*	150.6	36.0	302	176	33	0	[NC001865]
**Angiosperms**							
*Epifagus virginiana*	70.0	42.1	135	32	21	2 inverted	[NC001568]
*Nicotiana tabacum*	155.9	42.5	299	109	37	2 inverted	[NC001879]
**Euglenids**							
*Euglena longa*	73.3	32.0	135	56	28	3 tandem	[NC002652]
*Euglena gracilis*	143.2	50.9	118	88	40	3 tandem	[NC001603]
**Containing secondarily acquired red algal plastids**							
*Plasmodium falciparum*	34.7	4.7	24	34	34	2 inverted	[X95275, X95276]
*Toxoplasma gondii*	35.0	5.9	28	32	33	2 inverted	[NC001799]
*Eimeria tenella*	34.8	4.9	26	32	33	2 inverted	[NC004823]
*Theileria parva*	39.6	16.7	95	46	25	0	[AAGK01000009]
*Odontella sinensis*	119.7	15.6	107	146	29	2 inverted	[NC001713]

* the *Prototheca *genome is unfinished and its size is estimated from a restriction map [17], while the percentage non-coding DNA and mean intergenic distance were calculated from available sequences, constituting about half the genome.
